# Hyperreflective Choroidal Foci: A Comprehensive Review

**DOI:** 10.18502/jovr.v20.18397

**Published:** 2025-11-13

**Authors:** Mona Amer, Scott Sonne, Niloofar Piri

**Affiliations:** SSMHealth Medical Group, Department of Ophthalmology, School of Medicine, Saint Louis University, St. Louis, MO, USA

**Keywords:** Age-related Macular Degeneration, Biomarkers, Diabetic Macular Edema, Hyperreflective Choroidal Foci, Optical Coherence Tomography, Stargardt Disease

## Abstract

Hyperreflective choroidal foci (HCF) are a finding on optical coherence tomography that may serve as a biomarker in various retinal and choroidal pathologies. These discrete hyperreflective spots, identified in various layers of the choroid, have been linked to inflammatory, vascular, and degenerative conditions. This review examines the clinical significance, histopathological correlation, and implications of HCF in various diseases, including diabetic retinopathy, age-related macular degeneration, Stargardt disease, choroideremia, Vogt-Koyanagi-Harada disease (VKH), idiopathic posterior uveitis, retinitis pigmentosa, and central serous chorioretinopathy (CSR), as well as non-pathological states. Although further studies are required to validate the findings in each pathology described herein, HCF may be used as a background prognostic marker of disease progression and therapeutic response, albeit with caution.

##  INTRODUCTION

Hyperreflective foci are optical coherence tomography (OCT) findings first described by Coscas et al in 2009, in the retinas of patients with exudative age-related macular degeneration (AMD).^[1,2]^ It has since been described in many retinal pathologies. More recently, hyperreflective choroidal foci (HCF) have been described as discrete hyperreflective round spots that are at least as reflective as the RPE layer and are usually 10–50 microns in diameter.^[3]^ HCF have been reported in diabetic retinopathy, advanced AMD with geographic atrophy, posterior uveitis, as well as on optical coherence tomography angiography (OCTA) in eyes without known pathology. As with hyperreflective retinal foci (HRF), the substance of the hyperreflective signal of HCF is incompletely understood. Discordant histopathologic correlates have been postulated, as direct sampling of these microscopic foci *in vivo* is not feasible. Aside from Vogt-Koyanagi-Harada disease (VKH), every pathological state has been associated with more advanced pathology.

The purpose of this review is to synthesize what is known about HCF, including the findings in disease states known to have HCF, the implications in those disease states, the proposed etiology of HCF, and the opportunity for further investigations.

##  METHODS

A PubMed search was conducted using the terms “Choroidal Hyperreflective Foci”, “Hyperreflective Choroidal Foci”, “Hyperreflective Choroidal Dots”, and “Choroidal Hyperreflective Dots”. The articles included provided important insights into the understanding of HCF. Additionally, references were identified within these articles.

##  RESULTS

Articles on HCF were found to be published in nine pathological states as well as normal eyes. The pathologies included diabetic macular edema (DME), geographic atrophy (GA), Stargardt disease (SD), choroideremia, VKH, idiopathic posterior uveitis, retinitis pigmentosa, and central serous chorioretinopathy (CSR). In all these states, except for normal eyes and VKH, the presence of HCF is predictive of more advanced disease.

HCF has been best documented in diabetic retinopathy with DME, with multiple associations noted.^[4,5,6]^ The presence of HCF was found to be correlated with worse best corrected visual acuity (BCVA). In a study of 119 eyes with DME, eyes with HCF had a mean BCVA of 1.18 LogMAR (Logarithm of the Minimum Angle of Resolution) compared to 0.3 LogMAR in eyes without HCF.^[5]^ In another study of 43 eyes with treatment-naive non-proliferative diabetic retinopathy (NPDR) with DME, eyes with HCF had a mean BCVA of 0.59 LogMAR at initial presentation compared to 0.38 LogMAR in eyes without HCF.^[6]^

HCF have been associated with increased central foveal thickness and proliferative disease. Roy et al found that eyes with HCF had a mean central foveal thickness of 507 μm, while eyes without HCF had a mean CFT of 350 μm. Additionally, eyes with HCF are more likely to have PDR (58%) than those without HCF (33%).^[5]^

Limited information has been published pertaining to HCF in AMD.^[2,7,8,9,10]^ Borrelli et al studied the presence and number of HCF in 40 patients with GA.^[9]^ This study highlighted that HCF are easily identifiable in AMD by documenting their abundant presence in 100% of eyes with GA. HCF were mainly found near Bruch's membrane or the edges of blood vessels and were largely localized to the choriocapillaris and Sattler's layer. Foci found in the anterior choroidal layers appeared round, while those in the posterior choroidal layers were more oval. In this study, more HCF were identified in areas of complete RPE and outer retinal atrophy (cRORA).

**Figure 1:**
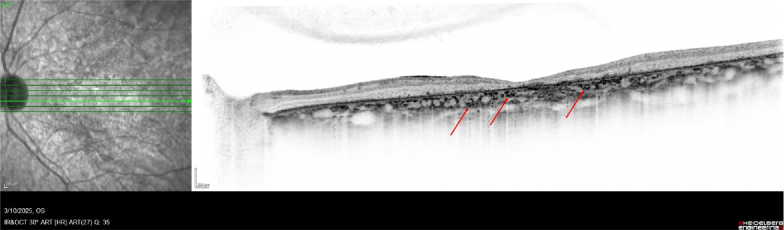
Spectral-domain optical coherence tomography through the macula showing outer retinal atrophy and hyperreflective choroidal foci (red arrows) in Stargardt disease.^[3]^

Multiple studies have reported a 100% incidence of HCF in eyes with SD [Figure 1].^[3,11,12]^ Similar to AMD, HCF in SD were most densely observed in the choriocapillaris and Sattler layers.^[3]^ Also similar to AMD, HCF in SD were round in superficial choroidal layers and were oval-shaped in deeper layers.^[3]^ There was a negative correlation between the number of HCF and visual acuity in SD, specifically the presence of HCF in the choriocapillaris and Sattler's layer.^[3,12]^ There was a positive correlation between disease duration and the number of HCF in either the choriocapillaris or Sattler's layer.^[3,12]^ A weak positive correlation was also found between HCF and subfoveal choroidal area, which is the thickness of the choroid measured between the RPE–Bruchs membrane complex and the choroidal–scleral junction.^[13]^ Additionally, higher numbers of foveal HCF were correlated with decreased central macular thickness and RPE atrophy.^[12]^ Higher numbers of HCF were seen in the pathological edges of atrophy in SD.^[11,12]^

Romano et al investigated the presence of hyperreflective foci in 10 patients with genetically proven choroideremia and 10 matched control patients.^[14]^ This study found statistically more HCF in patients with choroideremia than in healthy patients. It also revealed that these HCF were predominantly located in the pathological border of chorioretinal atrophy. Furthermore, choroidal thickness was negatively correlated with HCF density in the healthy border of areas of chorioretinal atrophy. HCF was also negatively correlated with the size of the spared central island.

In a study of 61 eyes with CSR, HCF were significantly higher in acute CSR than in chronic CSR. Additionally, the presence of HCF exhibited a strong inverse relationship with patients' age, and the number of HCF was negatively correlated with subfoveal choroidal thickness (SFCT). A further subgroup analysis of acute and chronic CSR was conducted. In acute CSR (*n* = 32), the presence of HCF was negatively associated with age and SFCT. In contrast, the presence of HCF in chronic CSR (*n* = 29) was not correlated with age but was inversely correlated with central macular thickness, spherical equivalent (the higher the spherical equivalent, the lower the number of HCF), SFCT, and height of neurosensory retinal detachment. Interestingly, the quantity of HCF was not correlated to any of the above parameters in acute CSR.^[15]^

In another study, eyes that had recovered from an acute episode of CSR had less HCF when compared to their previously active disease state, as well as in comparison to healthy fellow eyes and control eyes.^[16]^ HCF may represent exudates that form from cellular extravasation due to increased hydrostatic pressure and choroidal permeability. Authors in this study theorize that HCF are a function of choroidal fluid dynamics, whereby in the recovery phase of CSR, delayed resolution of choroidal stromal edema causes a dilution effect of pre-existing HCF remaining from the active phase and, as a result, decreased density of HCF.^[16,17]^

In contrast with other disease states, there are fewer HCF in VKH. In a study by Kim et al, they measured a decrease in HCF in eyes with VKH and sunset glow fundus compared to eyes with VKH without sunset glow fundus and eyes of healthy controls.^[18]^ Additionally, in eyes with VKH, a significant negative correlation was found between the degree of fundus pigmentation and the number and density of HCF.^[18]^ In another study, less HCF were observed in all stages of diseased eyes compared to controls.^[19]^ Additionally, HCF were found to have a strong negative correlation with fundus pigmentation at the fovea and the nasal parafovea in VKH.^[18]^ Enhanced depth imaging OCT was used to evaluate the choroid in patients with idiopathic posterior uveitis and panuveitis.^[20]^ Discrete HCF were seen in 35% of patients initially and at 6-month follow-up. Hyporeflective foci were also observed in the choroid in a similar proportion of patients (30%). These findings, however, were not correlated with visual acuity or disease severity.

HCF were present in a large percentage of patients in a study of retinitis pigmentosa (63%) and were found to occur more frequently in patients with thick choroid.^[21]^ HCF were shown as a potential biomarker of visual acuity, disease progression, and severity. Patients with HCF in the central outer retina, peripheral outer retina, or the choroid, or multiple areas, had worse visual acuity.^[21]^ The presence of hyperreflective foci in all three of the aforementioned areas was correlated with faster disease progression. In another study, HCF were positively correlated with the choroidal vascular index, although they were not associated with visual acuity or disease severity.^[13]^

Multiple studies have attempted to map the location of HCF on OCTA in eyes without pathology. One study found a greater HCF density near the central macula, and another study found that the quantity of HCF was positively correlated with the underlying stromal component of the choroid and negatively correlated with the subfoveal choroidal vascularity index. This indicates that HCF are not always pathological, and may represent a normal cellular component.^[17]^

HCF and HRF have been associated with choroidal neovascularization (CNV) due to angioid streaks. One prospective study examined retinal and choroidal hyperreflective foci and compared a control group of eyes with angioid streaks but without CNV with the study group having both angioid streaks and CNV. In a subgroup analysis, HCF were shown to be more abundant 1 month prior to the recurrence of fluid when compared to other times without fluid. The authors noted that this may be a useful predictive biomarker for recurrence of fluid.^[22]^

##  DISCUSSION

Limited histopathological studies, as well as the myriad of disease entities presenting with HCF, have left the subject of HCF open to debate. Among the published articles, theories about the origin of HCF frequently disagree, although those with similar disease states tend to have more compatible theories.

Studies of DME have found that in eyes with greater HRF, concentrations of IL-1β, CD14, and IL-6 are increased, indicating a correlation with inflammatory states.^[23]^ Bolz et al demonstrated the likely contribution of lipid exudation to HRF.^[1]^ It has been proposed that HRF are activated microglia that are resident within the retina (more likely to be found within the inner retina), possibly containing extravasated lipid. Microglial migration through the ELM is thought to be the mechanism of hyperreflective foci in the outer retina and the choroid. With disruption of Müller cell footplates, microglia are thought to migrate toward the outer retina and later the choroid.^[2,24]^ Saurabh et al found the ELM to be intact in 100% of eyes with only HRF as compared to 8.3% of eyes with HRF and HCF.^[6]^ Roy et al similarly published that only 35% of eyes with HCF had an intact ELM and EZ, compared to 98% of eyes without HCF.^[5]^ Notably, HCF without HRF have not been observed, indicating further that a sequence of migration through ELM toward choroid is plausible.

In idiopathic uveitis, both hyporeflective and hyperreflective foci were noted to be common in up to one-third of cases, although the authors did not explain their presence. Immunological cells or extravasation of cellular material are both plausible explanations in the context of uveitis and DME.^[20]^

Regarding GA, one theory suggests that HCF may be the result of posterior migration of disrupted RPE cells in areas of cRORA. Through histopathology, it has been demonstrated by Curcio et al that RPE cells migrate into the outer retina, making some contribution to the appearance of HRF (some demonstrated shadowing while others did not).^[8]^ These RPE cells have been theorized to also migrate toward the choroid, as they have been shown within Bruch's membrane, but there is no definitive evidence that they migrate past Bruch's membrane. Borrelli et al rather postulated that the presence of HCF in GA is a factor in unveiling of the non-pathological components of the choroidal stroma through increased signal transmission, as demonstrated in normal, non-pathological eyes.^[9,17,25]^

In Stargardt disease, it was proposed that lipofuscin material from the outer retina migrates into the choroid, although the authors could not rule out HCF findings due to improved visualization of the natural choroid. It was demonstrated that concomitant clearing of lipofuscin deposits within the outer retina occurred, along with an increase in HCF, in individuals with more advanced disease states. Similarly, in retinitis pigmentosa and choroideremia, HCF have been thought to be accumulations of migrated RPE cells with lipofuscin granules, most commonly found at the border of macular atrophy.^[14,21]^ As these diseases are not inflammatory, this is naturally in contrast to the theory of inflammatory cells representing HCF.

Hanumunthadu et al theorized that, as the driver of pathology is different, so is the mechanism of HCF in CSR from the immunological theory in DME. As alteration of choroidal structure leads to an increase in vascular permeability resulting in extravasation of serous fluid, authors posit that the HCF represent extravasated cellular debris.^[15]^

Kim et al suggest that the decrease in HCF in VKH with sunset glow fundus may be due to the loss of melanin granules in choroidal melanocytes. The authors demonstrated that diminished HCF was correlated with the degree of depigmentation of the choroid (*P* < 0.001). In eyes with sunset glow fundus, there were significantly fewer HCF than in other stages of VKH. Similarly, Fong et al demonstrated decreased presence of HCF in the inner choroid in patients with VKH compared with age-matched healthy subjects.^[19]^ Hyperreflective foci within the choroid of normal eyes have been theorized to represent pigments from large melanocytes in choroidal stroma, which contain many melanosomes. This aligns with the previously mentioned theory that decreased HCF in eyes with sunset glow fundus is likely related to a decrease in melanocytes or melanosomes.^[25]^

Migration of inflammatory cells pervades theories in DME and uveitis. Migration of RPE is endorsed in RP, choroideremia, and plausibly GA. Migration of lipofuscin is the prevailing theory in Stargardt disease. These hypotheses are consistently based upon chemotaxis of either cellular material or intact cells from the damaged retina toward the choroid.^[26,27,28]^

To date, multiple theories have been presented to explain HCF in various disease states. Many authors have attempted to find a unifying theory surrounding the substance of hyperreflective foci in retinal and choroidal pathology. Although HCF may be the final product of quite different pathological states, it may also be an image of varying dense cells, including but not limited to the migration of microglia, macrophages, RPE cells, lipofuscin, or natural pigmentation of the choroidal stroma.

##  SUMMARY

HCF likely represent an important biomarker in many retinal and choroidal diseases. Their presence and distribution offer valuable insights into disease state and progression. Future research may help elucidate the etiology of HCF through histopathological correlates with donor eyes. Other applications may involve progression analysis on OCT to indicate changes in HRF and HCF from baseline. Another application may be similar to traditional imaging techniques, such as MRI and CT, in which gray-scale analysis provides feedback about the substance of the images. OCT density studies of various cell types within the retina may be applied to detect what these foci represent quantitatively.^[29]^

##  Financial Support and Sponsorship

None.

##  Conflicts of Interest

None.
